# Measure on innovation efficiency of China's pharmaceutical manufacturing industry

**DOI:** 10.3389/fpubh.2022.1024997

**Published:** 2022-11-24

**Authors:** Shen Zhong, Shuqi Liang, Yuxin Zhong, Yunying Zheng, Fengjun Wang

**Affiliations:** ^1^School of Finance, Harbin University of Commerce, Harbin, Heilongjiang, China; ^2^Department of Neurology, The Fourth Affiliated Hospital of Harbin Medical University, Harbin, Heilongjiang, China

**Keywords:** pharmaceutical manufacturing, innovation efficiency, super network SBM model, Global-Malmquist index, DEA

## Abstract

Pharmaceutical manufacturing industry is an important industry to ensure human life safety. The innovation efficiency is a significant factor to stimulate the development of pharmaceutical manufacturing industry. At present, there are few studies on the innovation efficiency of pharmaceutical manufacturing industry. To fill this gap, this paper estimates the innovation efficiency of China's pharmaceutical manufacturing industry in 23 provinces of China from 2010 to 2020 based on the super-network SBM model and Global-Malmquist index. The results show that: (1) From the perspective of efficiency of research and development stage (ERDS), the ERDS of China shows an increasing trend, with the most prominent growth in the western region. (2) From the perspective of efficiency of economic transformation stage (EETS), although there are fluctuations in the EETS, the overall development is good. The EETS of the central region and western region is better than that of the eastern region. (3) By comparing the efficiency of the two stages, it is found that the change direction of the efficiency of the two stages is not necessarily the same in some provinces.

## Introduction

The pharmaceutical manufacturing industry is one of the high-tech industries. Its development not only represents the China's level of Sci. & Tech and economic strength (1), but also is an important industry to ensure human health and life (2). According to data released by the National Bureau of Statistics, over the past two decades, Chinese per capita consumption expenditure on health care has maintained a growth level of more than 10%, exceeding the growth rate of Chinese per capita consumption level (8%). In 2021, the growth rate of Chinese per capita consumption expenditure on health care reached a new high of 14.8%, which means the demand for national medicine has always remained stable. As a knowledge-intensive industry (3), on the one hand, according to different specific diseases, the demand for medicine is diversified. For example, the discovery of unknown diseases and the resistance to existing drugs caused by the antibiotics abuse are major challenges for the medical manufacturing industry, which promote the continuous innovation of pharmaceutical enterprises. On the other hand, the launch of a patented new drug can often monopolize or even open up a market segment, and the new products can bring huge profits to developers (4). So innovation efficiency is an important factor to promote the transformation and upgrading of the pharmaceutical manufacturing industry and improve its core competitiveness (5).

However, the innovation ability of China's pharmaceutical manufacturing industry is weak. According to the list of top 50 global pharmaceutical enterprises, which published by PharmExce in 2021, only four Chinese pharmaceutical enterprises are selected. The top 50 pharmaceutical enterprises are mainly from the United States, Japan, Germany and other developed countries. This is mainly because the pharmaceutical manufacturing industry of China started late and its independent innovation ability is weak (6). Innovation technology is still mainly imported from abroad (1, 7), and most of the pharmaceutical products are generic drugs (8) The innovation efficiency of China's pharmaceutical manufacturing industry needs to be improved urgently. Two issues are as follows: First, the current researches on the innovation efficiency of pharmaceutical manufacturing industry are mostly focused on the efficiency evaluation of a single stage, that is, only the input of various funds and manpower and the output of sales revenue of new products are considered. While the innovation process of the pharmaceutical manufacturing industry is a closely linked dual stage. The input in the first stage is scientific research funds and human resources, and the output is the number of patent applications. At the same time, the number of patent applications is the input in the second stage, and the final output is the sales revenue of new products (9). Two stages can open the “black box,” consider the internal structure, more objectively and accurately evaluate the efficiency gap of each stage and analyze the reasons, targeted to improve the efficiency of innovation. Secondly, previous studies did not take the unbalanced development of regional pharmaceutical innovation into account. Due to the vast territory, abundant resources and large population of China (1). The distribution, foundation and structure of the pharmaceutical industry in various regions are different, and there are interval differences, which cannot form the overall development of technical resources and human resources (10). Thus hindering the progress of the innovation ability of the pharmaceutical manufacturing industry. Therefore, in combination with these two aspects, the efficiency of the first and second stages of the pharmaceutical manufacturing industry in different regions of China is different. Accurately grasping the efficiency of different stages in different regions can improve the innovation efficiency of China's pharmaceutical manufacturing industry more pertinently, which has important theoretical and practical significance for improving the imbalance of regional development of China's pharmaceutical manufacturing industry.

Therefore, this paper has the following three contributions: First, this paper is the first study on the innovation efficiency of China's pharmaceutical manufacturing industry, and the research topic is innovative. Second, this paper uses the super-efficiency network SBM model and GM index to divide the innovation process of the pharmaceutical manufacturing industry into two stages. At the same time, it combines with the problem of regional heterogeneity to explore the differences of different stages in different regions, which provides a new idea for related research. Third, using the latest available data, this paper collected data from 23 provincial levels in China from 2010 to 2020 to analyze the efficiency of pharmaceutical manufacturing in different stages in different regions, so as to understand the actual situation of innovation efficiency of pharmaceutical manufacturing in China.

The rest of this paper is arranged as follows: The second part is the review of the existing papers. The third part is the research methods, cited data, and the empirical analysis. The conclusions and relevant policy suggestions are presented in the fourth and fifth parts respectively.

## Literature review

It has always been a hot topic in academic research to examine technological innovations. Many studies on its measurement have been conducted by scholars at home and abroad, which has also laid a solid foundation. However, there are some deficiencies in the research. This paper will supplement the deficiencies.

Scholars from different countries have made great contributions to the innovation of pharmaceutical manufacturing industry from different perspectives. Cai et al. (11) and Chen et al. (12) showed that the innovation capability of the pharmaceutical manufacturing industry in the United States, the European Union and Japan ranked first in the world. Scherer (13) empirically analyzed American pharmaceutical companies and found that high innovation brings high performance and effectively avoids profit damage caused by competition. It is concluded that the emphasis on innovation will be profitable. Keyhani et al. (14) also came to the same conclusion. Hashimoto and Haneda (15) used Malmquist index (MI) to study the R&D efficiency of Japanese pharmaceutical industry from 1991 to 2000. They found that the R&D efficiency was declining. Although Japanese medicine has become less innovative every year, but Hshimoto and Haneda (16) believed that the innovation ability of Japanese medicine was still in the leading position in the world. At present, there are abundant studies on the innovation efficiency of medical manufacturing industry in developed countries such as the United States and Japan, but there are relatively few studies on the innovation efficiency of Chinese medical manufacturing industry. Cai et al. (11) believed that Chinese pharmaceutical enterprises have strong imitation ability, but weak innovation ability. How to improve innovation efficiency? Wang et al. (17) included independent research and development, innovation performance and technology accumulation into the same research framework, and found that R&D personnel input and technology purchase can promote innovation efficiency, while technology introduction and technology adaptation have no significant impact on innovation efficiency. Lai et al. (18) used DEA model to analyze the innovation performance of pharmaceutical manufacturing industry in 28 provinces of China and found that U-shape trap exists. For regions with low innovation performance in the trap, they should seek new technology and new industry transformation instead of blindly increasing investment intensity.

There are two problems in the above research: First, most of the literature is an overall study of Chinese pharmaceutical enterprises, which does not consider the issues of specialized aggregation and diversified aggregation. There are differences in cooperative innovation intensity and industrial structure among different regions (10), which have different impacts on their innovation efficiency. Therefore, it is very necessary to study the innovation efficiency of pharmaceutical manufacturing industry by region to form the overall development of technical resources and human resources and improve the innovation efficiency of pharmaceutical enterprises (1). There are also differences in innovation efficiency in different regions. Second, most current studies regard the innovation process as a single stage, that is simply investigate the input of human resources and the output of income. However, there is a problem that the innovation process of pharmaceutical manufacturing industry is characterized by obvious dual stages (19). Generally, it can be divided into the research and development stage and economic transformation stage (20). In the research and development stage , pharmaceutical enterprises continue to invest scientific research funds and human resources to develop patents. Patents are not only the output of the research and development stage, but also the input of economic transformation stage. In this stage, patents are converted into new products to obtain sales revenue (21). Therefore, to open the “black box” of the innovation process of the pharmaceutical manufacturing industry, we need to consider the internal structure of the industry, where allows us to more objectively assess the efficiency gap of each stage, and analyze the reasons, so as to improve the efficiency of innovation in a target manner (9).

Combining the above two problems, selecting an appropriate model is also the focus of our study. Most previous studies were limited to the traditional stochastic frontier analysis (SFA) in parametric form and data Envelopment analysis (DEA) in non-parametric form. Although the SFA model is also widely used in the evaluation of innovation efficiency (22–24), but since SFA needs to construct a specific production function form, it will be subject to subjective influence of the author. At the same time, if there are many input-output indicators, the evaluation results of SFA are prone to deviate from the actual situation (25). Compared with SFA model, DEA does not need to set functional form and is suitable for evaluating decision making units with multi-input and multi-output structure (26). Therefore, DEA model is more suitable for the innovation efficiency evaluation of pharmaceutical manufacturing industry (27). However, these DEA models regarded DMU as a “black box” and did not consider innovation as an organic process (28). For this reason, Seiford and Zhu (29) directly applied the standard DEA model to the two sub-stages respectively. Kao and Hwang (30) obtained the overall efficiency by multiplying the efficiency of the two sub-stages. However, these studies split innovation into two independent stages, failed to consider or solve the conflicts between the two stages caused by intermediate variables, and ignored the correlation between the stages and the integrity of innovation activities (31). In order to solve this problem, this paper adopts the super network SBM model (32). When evaluating the innovation efficiency of pharmaceutical manufacturing industry, this model can better evaluate the sub-stage efficiency of pharmaceutical manufacturing industry and compare and analyze the differences in sub-stage efficiency. At the same time, Global-Malmquist (GM) index is used to analyze regional heterogeneity. GM index is developed from the traditional Malmquist index (MI) (33). Compared with MI, GM index introduces population heterogeneity into the research process which is more suitable for regional difference analysis (34).

Based on this, this paper selects the super network SBM model and GM index, and divides the country into three regions: eastern region, central region and western region. At the same time, the innovation process of pharmaceutical manufacturing industry is divided into the research and development stage and economic transformation stage. The analysis of the different stages' efficiency in different regions is convenient to understand the actual situation and has important theoretical and display significance for improving the innovation efficiency of the pharmaceutical industry.

## Model construction and data

### Super network SBM model

There is a problem of slack variable in the traditional DEA model. For purpose of solving this problem, the SBM model was proposed (35), as follows:


(1) $$\begin{array}{l} min\rho =\frac{1-\frac{1}{M}\sum_{i=1}^{M}\frac{s_{i}^{-}}{x_{i0}}}{1+\frac{1}{N}\sum_{r=1}^{N}\frac{s_{r}^{+}}{y_{r0}}} \end{array}$$

(2) $$s.t.\{\begin{array}{c} x_{0}=X\lambda +s^{-}\\ y_{0}=Y\lambda -s^{+}\\ \lambda \geq 0,s^{-}\geq 0,s^{+}\geq 0 \end{array}$$

The basic SBM model regards the innovation process is considered to be a “black box,” which is simply input into the “black box” and then output from the “black box,” but the specific process in the “black box” has not been analyzed. In order to solve this shortcoming, based on the network SBM model, a two-stage model of innovation achievements in pharmaceutical manufacturing industry is established (32). Under the circumstance of this model including the advantages of the basic SBM model, the network SBM model is:


(3) $$\rho_{0}^{*}=\frac{\sum_{k=1}^{K}\omega^{k}[1-\frac{1}{m_{k}}\sum_{i=1}^{m_{k}}\frac{s_{i}^{k-}}{x_{i0}^{k}}]}{\sum_{k=1}^{K}\omega^{k}[1+\frac{1}{r_{k}}\sum_{r=1}^{r_{k}}\frac{s_{r}^{k+}}{y_{r0}^{k}}]}$$

(4) $$s.t.\{\begin{array}{c} x_{0}^{k}=X^{k}\lambda^{k}+s^{k-}\\ y_{0}^{k}=Y^{k}\lambda^{k}-s^{k+}\\ Z^{\left(k,h\right)}\lambda^{h}=Z^{\left(k,h\right)}\lambda^{k}\\ e\lambda^{k}=1\\ \lambda^{k}\geq 0,s^{k-}\geq 0,s^{k+}\geq 0 \end{array}$$

In the network SBM model mentioned above, the production capacity of each DMU is compared with the optimal frontier. When the result is 1, the DMU is effective. Otherwise, it is ineffective (36). However, this model cannot distinguish the differences between effective DMUs. In the super network model, the differences between effective DMUs can be distinguished. The super network SBM model is as follows for this goal:


(5) $$\begin{array}{l} \rho =\underset{\lambda^{k}s^{k-},s^{k+}}{\min}\frac{\sum_{k=1}^{K}\omega^{k}[1-\frac{1}{m_{k}}\sum_{i=1}^{m_{k}}\frac{s_{i}^{k-}}{x_{i0}^{k}}]}{\sum_{k=1}^{K}\omega^{k}[1+\frac{1}{r_{k}}\sum_{r=1}^{r_{k}}\frac{s_{r}^{k+}}{y_{r0}^{k}}]} \end{array}$$

(6) $$s.t.\{\begin{array}{c} x_{0}^{k}=X^{k}\lambda^{k}+s^{k-},k\neq 0\\ y_{0}^{k}=Y^{k}\lambda^{k}-s^{k+},k\neq 0\\ Z^{\left(k,h\right)}\lambda^{h}=Z^{\left(k,h\right)}\lambda^{k}\\ e\lambda^{k}=1\\ \lambda^{k}\geq 0,s^{k-}\geq 0,s^{k+}\geq 0 \end{array}$$

The efficiency of stage k is:


(7) $$\begin{array}{l} \rho_{k}=\frac{1-\frac{1}{m_{k}}(\sum_{i=1}^{m_{k}}\frac{s_{i}^{k-}}{x_{i0}^{k}})}{1+\frac{1}{r_{k}}(\sum_{r=1}^{r_{k}}\frac{s_{r}^{k+}}{y_{r0}^{k}})}(k=1,2,\ldots ,K) \end{array}$$

In formula (3–5), K rerpresents the stages, as in k = 1, 2, …, K. ω^*k*^is the weight value of stage k, and $\Sigma_{k=1}^{K}\omega^{k}=1$,ω^*k*^≥0(∀*k*), m_k_ and r_k_ are the indicator quantities of input and output in stage K respectively. Z^(k, h)^ is the intermediate output between stage k and stage h. x_0_ and y_0_ are the m-dimensional input variables and p-dimensional output variables of the evaluated decision-making unit DMU_0_ respectively. X and Y represent the input and output matrix of the decision-making unit. s^k+^and s^k−^are slack variables of output and input, respectively.

### Global-Malmquist index and its decomposition

Pastor and Lovell (34) proposed a method of calculating Malmquist index known as global-Malmquist. This system uses the sum of each period as the reference set, or in other words, the common reference set of each period is as follows:


(8) $$\begin{array}{l} P^{G}\left(x\right)=P^{1}\left(x^{1}\right)\cup P^{2}\left(x^{2}\right)\cup \ldots \cup P^{T}\left(x^{T}\right) \end{array}$$

A single Malmquist index is calculated for each period using the same frontier. The MI in the GM index is:


(9) $$\begin{array}{l} MI_{t}^{t+1}=\frac{E^{g}\left(x^{t+1},y^{t+1}\right)}{E^{g}\left(x^{t},y^{t}\right)} \end{array}$$

The computing method of EC is as follows:


(10) $$\begin{array}{l} EC=\frac{E^{t+1}\left(x^{t+1},y^{t+1}\right)}{E^{t}\left(x^{t},y^{t}\right)} \end{array}$$

Frontier t+1 is proximity to the global frontier can be expressed as $\frac{E^{g}\left(x^{t+1},y^{t+1}\right)}{E^{t+1}\left(x^{t+1},y^{t+1}\right)}$. As the ratio increases, the frontier t+1 gets closer to the global frontier. B represents how close the frontier t is to the global frontier. As a matter of fact, the larger the ratio, the closer the frontier t is to the global frontier. As a result, frontier t+1 is expressed by the ratio of two ratios:


(11) $$\begin{array}{l} TC_{g}=\frac{\frac{E^{g}\left(x^{t+1},y^{t+1}\right)}{E^{t+1}\left(x^{t+1},y^{t+1}\right)}}{\frac{E^{g}\left(x^{t},y^{t}\right)}{E^{t}\left(x^{t},y^{t}\right)}}=\frac{E^{g}\left(x^{t+1},y^{t+1}\right)}{E^{t+1}\left(x^{t+1},y^{t+1}\right)}\frac{E^{t}\left(x^{t},y^{t}\right)}{E^{g}\left(x^{t},y^{t}\right)} \end{array}$$

It is possible decompose MI into efficiency change and technology change (37):


(12) $$\begin{array}{l} MI_{t}^{t+1}=\frac{E^{g}\left(x^{t+1},y^{t+1}\right)}{E^{g}\left(x^{t},y^{t}\right)}=\frac{E^{t+1}\left(x^{t+1},y^{t+1}\right)}{E^{t}\left(x^{t},y^{t}\right)}\\ \;\times \left[\frac{E^{g}\left(x^{t+1},y^{t+1}\right)}{E^{t+1}\left(x^{t+1},y^{t+1}\right)}\frac{E^{t}\left(x^{t},y^{t}\right)}{E^{g}\left(x^{t},y^{t}\right)}\right]=EC\times TC_{g} \end{array}$$

### Establishment of two-stage structure

The existing literature on the evaluation model of pharmaceutical manufacturing industry's innovation efficiency stays in a single stage mostly. Based on previous studies, this paper divides the single stage into research and development stage and economic transformation stage, which not only shows the respective input and output of each stage, but also links the two stages through intermediate indicators, considering the internal process of innovation.

The two-stage model of pharmaceutical manufacturing industry is as follows: The first stage is research and development stage. The second stage is economic transformation stage. Second stage input is first stage output. In the first stage, research and development stage, the input of full-time equivalent of R&D personnel and R&D expenses can not produce economic benefits directly, but will produce intermediate output. There are three input indicators: intramural expenditure on R&D expenses (X_1_), full-time equivalent of R&D personnel (X_2_) and expenditure on new products development (X_3_). The intermediate output is the number of invention patent applications (Z). In the second stage, economic transformation stage, the input of this stage is the output of the first stage, and the final output is the sales revenue of new products (Y). The intermediate output is transformed into the final products with economic benefits.

**Figure 1:**
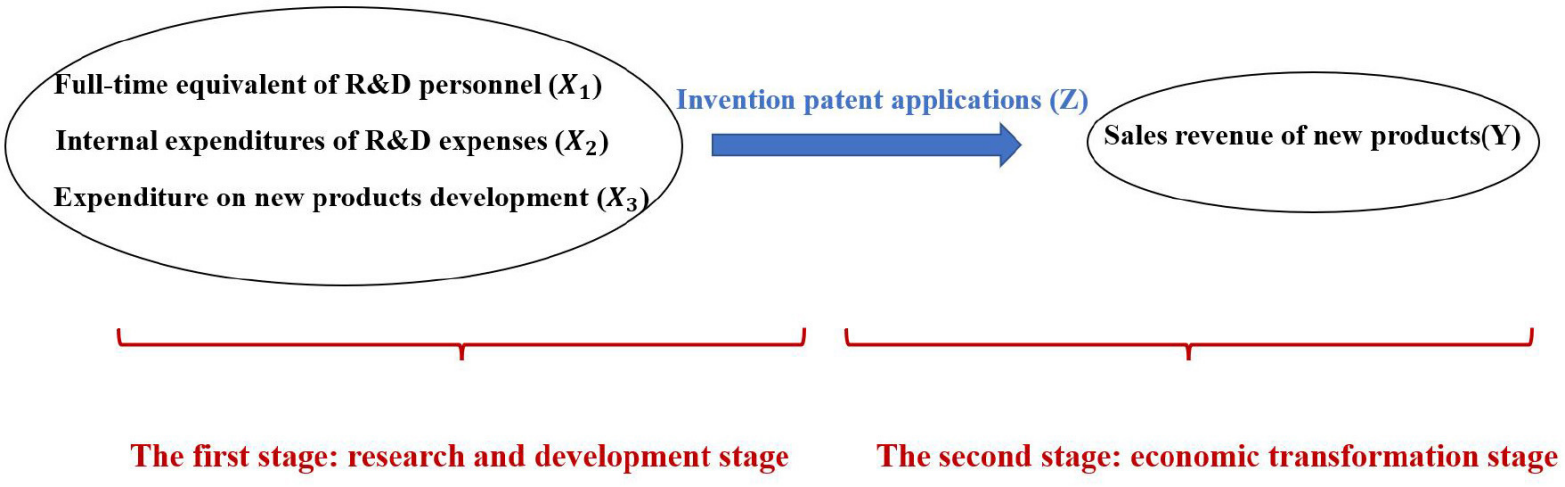
Innovation network structure of pharmaceutical manufacturing industry.

According to the selected input and output indicators, a specific efficiency model is established, as shown in Figure 1.

### Data and descriptive statistics

#### Index selection and data processing

##### Selection of indicators

In this paper, we construct a system of efficiency evaluation indexes of the research and development stage and the economic transformation stage for pharmaceutical manufacturing industry, respectively (Table 1). In the first stage, the input indicators are full-time equivalent of R&D personnel, internal expenditures of R&D expenses and expenditure on new products development. The output indicators are the numbers of invention patent applications. In the second stage, the input index is the invention patent applications, that is the output of the first stage. The output index is the sales revenue of new products. The meaning of indicators is as follows:

(1) Internal expense of R&D: R&D activities are the core of scientific innovation activities and the key points. Expenses for new product development, new processes and new technology development are included in the internal R&D expense. This indicator is a human input indicator, which reflects the human input of various regions for R&D activities.
(2) R&D personnel full-time equivalent: Human capital is an investment in innovation. It is an indicator used internationally for comparing human investment in Sci-Tech innovation. In other words, it refers to the total workload of full-time R&D personnel throughout the year who work 90 percent or more of the total working hours per year in R&D activities and the amount of work converted by personnel working part-time according to the actual working hours. This indicator is a human input indicator, which reflects the human input of various regions on research and development activities.
(3) Expenditure on new products development: It represents the capital expenditure of enterprises for new products, reflecting the fund support for new product development. This index is the financial investment index, reflecting the regional investment in new products.
(4) Numbers of invention patent applications: It refers to the innovation ability in this field. The more invention patent applications, the stronger the innovation ability in this field, and vice versa. In addition to the output index of the first stage, this index also represents the input index of the second stage, that is, the intermediate index, reflecting the amount of invention patents authorized in each region.
(5) Sales revenue of new products. During the enterprise's business period, it is the sales revenue generated by new products. The sales revenue of new products reflects the market acceptance. This index is the output index of economic benefits, which reflects the economic transformation ability of innovation achievements.

**Table 1:** Descriptive statistics of the indexes.

**Stage**	**Criterion layer**	**Index**	**Unit**
Research and development stage	Input	Full-time equivalent of R&D expenses	10,000 yuan
		Internal expenditures of R&D expenses	Man-year
		Expenditure on new products development	10,000 yuan
	Output	Invention patent applications	Piece
Economic transformation stage	Input	Invention patent applications	Piece
	Output	Sales revenue of new products	10,000 yuan

##### Processing of missing data

Due to the large span of the original data in this paper, involving many provinces and cities, and the loss of individual index data, this paper uses interpolation to make up for the missing data, that is, the average value of the data in the next 2 years of a year is used to replace the data in that year. P_t_ is used to represent the P index of a city in t year. If this value is missing, it can be replaced by the average value of the data of previous year and next year, that is, P_t_ = (P_t − 1_+P_t+1_) /2.

#### Data sources

In this article, we present data from the “China High-tech Industry Statistical Yearbook” for 2009 to 2020. To provincial administrative division unit, the pharmaceutical manufacturing industry is divided into 31 samples in this paper. In the process of data collection, it is found that the pharmaceutical manufacturing scale in Inner Mongolia, hainan, guizhou, Tibet, gansu, ningxia, related, qinghai, xinjiang, Taiwan and Macao is relatively small, and data is lack. Therefore, these provinces are excluded. The pharmaceutical manufacturing industry in the remaining 23 provinces was selected as the research object.

**Figure 2:**
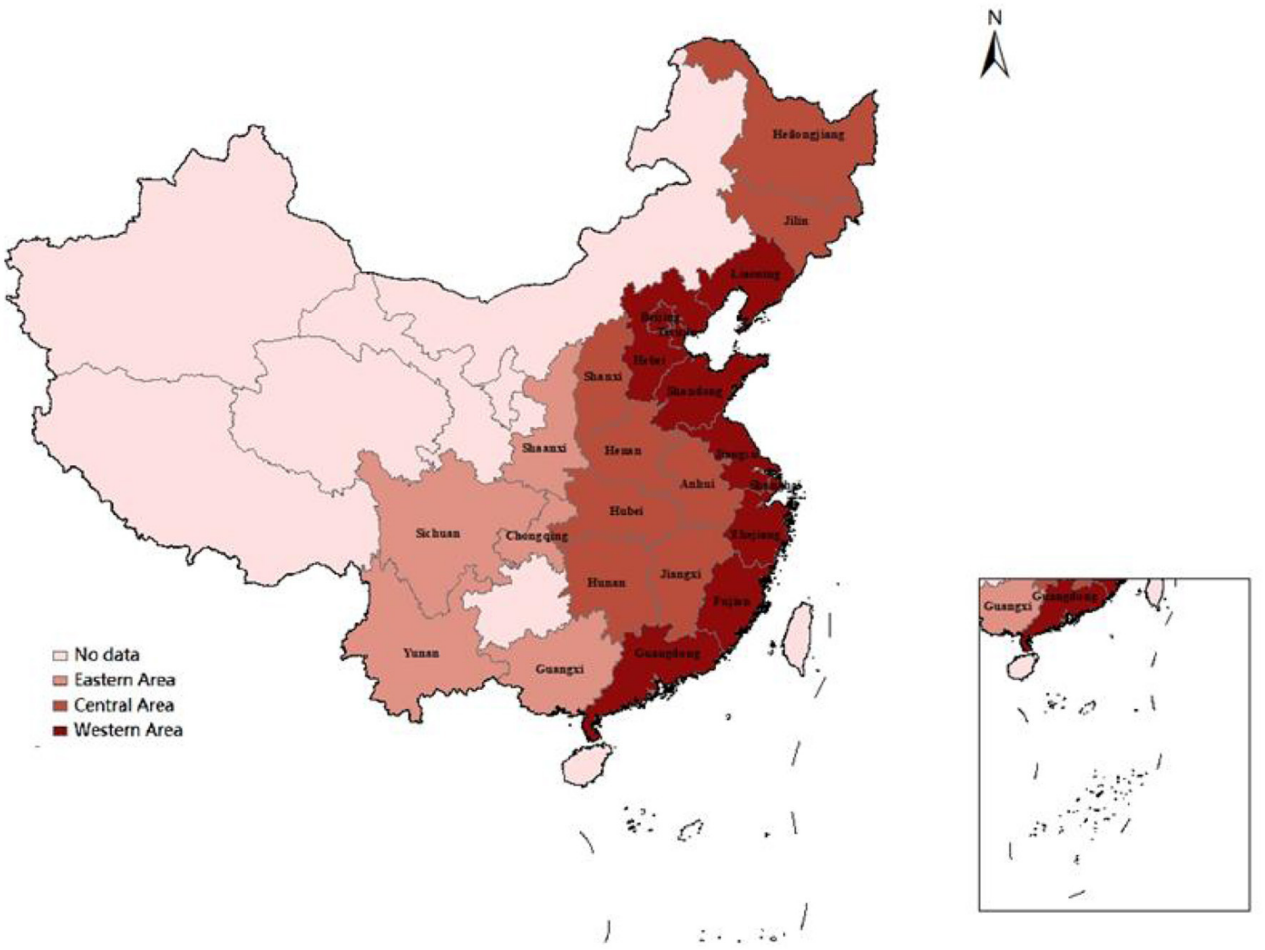
Division of eastern, central and western regions.

We divide these 23 provinces into three regions: the eastern region (Beijing, Liaoning, Tianjin, Hebei, Shanghai, Fujian, Zhejiang, Jiangsu, Shandong, Guangdong), the central region (Shanxi, Anhui, Jilin, Heilongjiang, Jiangxi, Henan, Hubei, Hunan), and the western region (Sichuan, Yunnan, Shanxi, Chongqing, Guangxi) (38). As shown in Figure 2.

## Efficiency evaluation of Chinese pharmaceutical manufacturing industry

In this paper, MaxDEA8.0 software was used to measure the efficiency of the research and development stage (ERDS) and the efficiency of economic transformation stage (EETS) in pharmaceutical manufacturing industry from the perspective of time and space.

### Research on the ERDS

#### Research on the ERDS from temporal perspectives

**Figure 3:**
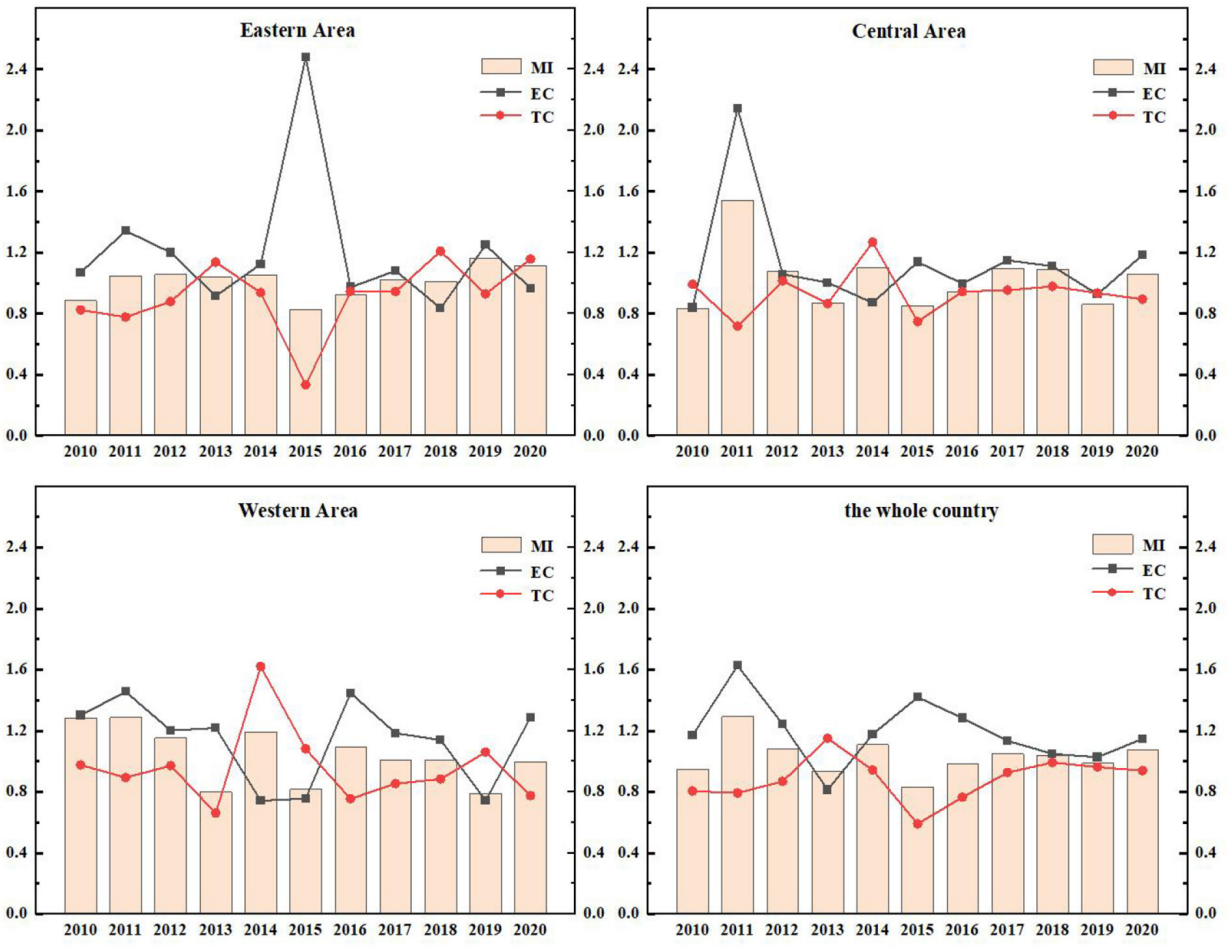
ERDS and its decomposition index in Chinese different regions from 2010 to 2020.

The ERDS reflects the ability of researchers and research funds to convert their input into patent output. Due to different geographical locations, transportation factors, development and openness, R&D capabilities are different in different regions. In Figure 3, the total factor productivity (TFP) of eastern China in 2010, 2015, and 2016 was 0.8869, 0.8301, and 0.9226 respectively. They were all < 1. The ERDS has declined, which can be increased by 11.31, 16.99, and 7.74%, respectively compared with the optimal distance level. Further study of these 3 years shows that EC and TC in 2016 are both < 1, which were 0.9733 and 0.9467, respectively. This shows that the technological change and efficiency change in 2012 have declined, resulting in a decrease in the efficiency of research and development this year. By observing the data of 2010 and 2015, scientific research found that the TC was < 1, indicating that the technology has regressed in the past 2 years, resulting in the improvement of scientific and technological research and development by 11.31 and 16.99%, respectively compared with the optimal distance level.

The TFP of the central region from 2010 to 2020 was 0.8331, 1.5435, 1.0773, 0.8720, 0.9412, 1.0964, 1.0881, 0.8627, and 1.0592 respectively. The data fluctuated. Among these years, there were short-term declines in 2010, 2013, 2015, 2016, and 2019, and the efficiency value was < 1. The rest of the years grossed positively. Further analyses of the data in the years that the ERDS decreased showed that the TC in these 5 years was < 1, which indicates that the main reason for the low ERDS was technological regression.

The TFP of the western region from 2010 to 2018 was 1.2857, 1.2882, 1.1521, 0.7995, 1.1944, 0.8179, 1.0961, 1.0110, 1.0087, 0.7912, and 0.9981, respectively. Except for the declines of 2013,2015, 2019, and 2020, the TFPs in other years are >1. The capacity of research and development has improved. During this period, the efficiency of research and development in 2011 was 1.2882, which is the highest. The EC and TC of 2011 were 1.4558 and 0.8933, respectively, which indicates that efficiency progress drives the efficiency of research and development.

During this period, the country's overall R&D efficiency has improved in all years except for a brief decline in 2010, 2013, 2015, 2016, and 2019, which is mainly attributed to the improvement in efficiency progress. In general, the research and development stage of China's pharmaceutical manufacturing industry has developed well. Because the state attaches importance to the innovation and development of the pharmaceutical manufacturing industry and increases the investment in human and financial resources, the ERDS has improved. However, there are still fluctuations in efficiency, which is largely related to the characteristics of this industry. First of all, the pharmaceutical manufacturing industry is a high-tech industry, which is knowledge-intensive and technology-intensive. It is very difficult to develop key technologies, and the rates of risk and failure are high. Secondly, the product changes quickly, but it is difficult to determine when it will be replaced.

**Figure 4:**
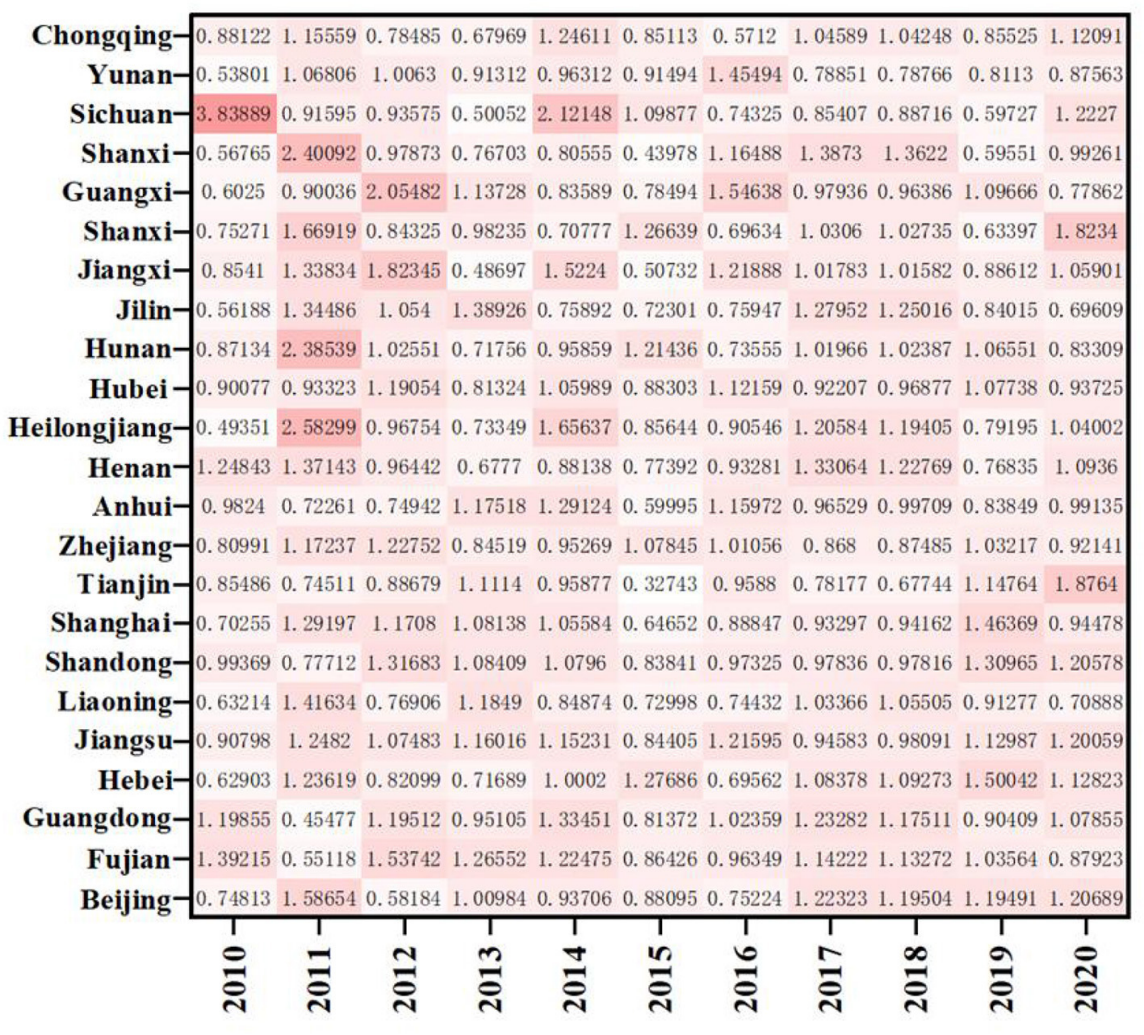
The ERDS in different provinces from 2010 to 2020.

As shown in Figure 4, except for Yunnan and Chongqing, the ERDS in the western region is < 1, and the ERDS in other provinces is >1, growing in a positive way. This is due to the implementation of the strategies of “industrial transfer” and “western development,” the western region has been narrowing its gap with advanced regions over the past few years, and the ability of ERDS has been improved. The ERDS in the eastern provinces is high, and the TFP is 1.0138. Because there are many developed cities in the eastern regions with sufficient scientific facilities, R&D personnel and capital investment, high degree of openness, and the convenient transportation. It will further attract more R & D talents and investment funds, and greatly improve level of R & D under these conditions. However, It is important to note that Guangxi, located in the western region, only showed a growth in 2012, 2013, 2016, and 2019 with the ERDS is >1 and the progress of technological research and development capacity. The rest of the years showed a state of regression. Compared with other west cities, Guangxi has the least internal expenditure input of R&D funds during this period, with an average value of 1736.88 million yuan. Therefore, even if the output was less, the ratio would be higher. In addition, when the input is less, the redundancy and waste will be relatively less, which improves the output efficiency to a certain extent.

#### Research on the ERDS from spatial perspective

**Figure 5:**
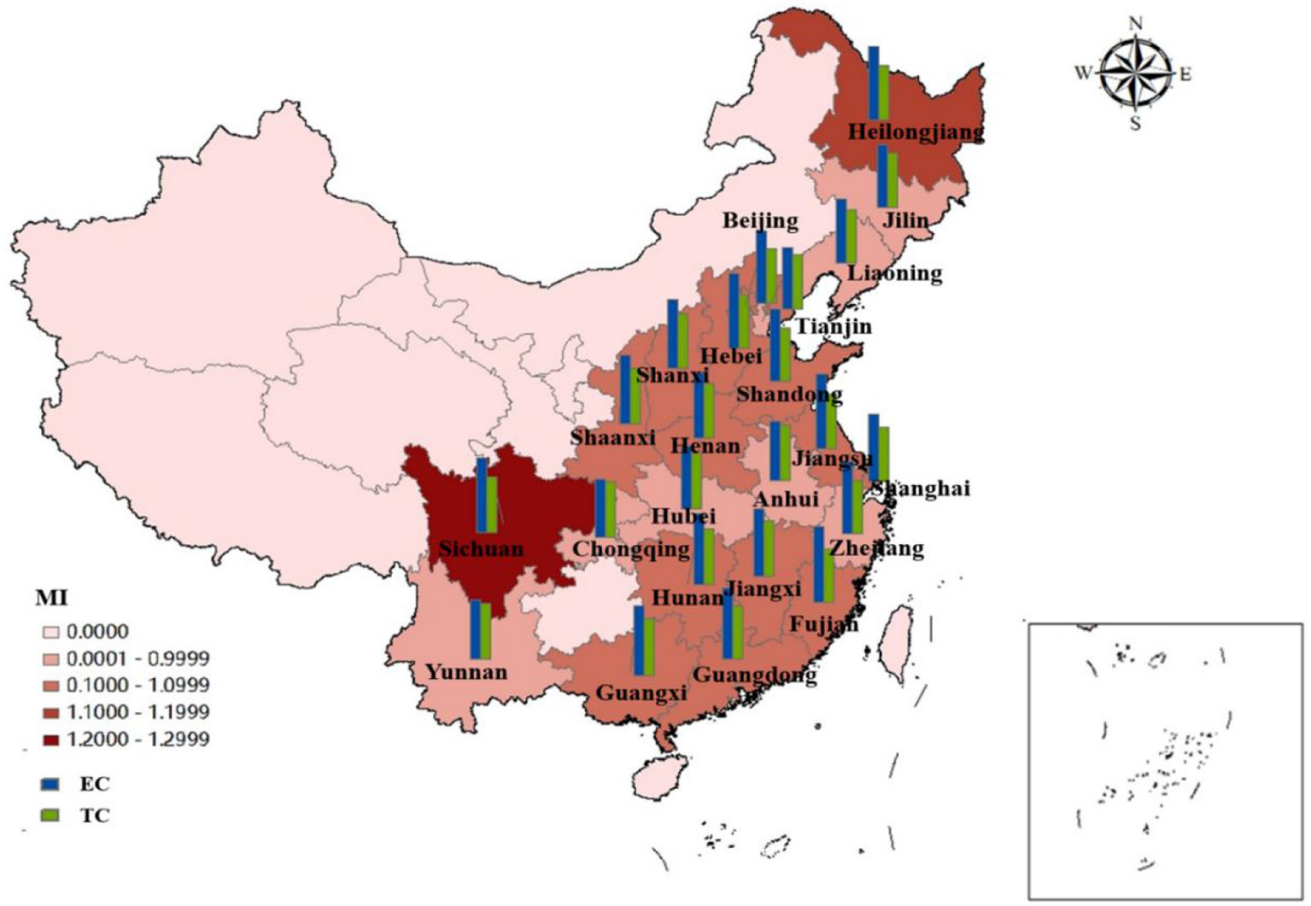
Average efficiency and its decomposition index of ERDS in 23 Chinese provinces from 2010 to 2020.

As shown in Figure 5, we divide these 23 provinces into three regions: the eastern region, the central region and the western region. By observing the efficiency and its decomposition index of the three regions, respectively, we can find that the ERDS in the three regions has been raised to varying degrees from 2010 to 2020, with an increase of 1.38, 3.01, and 4.03%, respectively. The ERDS in the western region has increased most significantly. From the figure we can see, efficiency progress has made a major contribution to improve the capacity of R&D in the western region. Among them, Sichuan (1.2469) was the best, because Sichuan has more colleges and universities and stronger scientific and technological research and development capabilities compared with other cities in the western region,. In the meantime, enterprises can obtain better opportunities for foreign exchange and have stronger R&D capabilities. The central region takes the second place. In Anhui (0.9525), Hubei (0.9825) and Jilin (0.9688), the ERDS is below 1, which is less than the national average. Technological regression limits the improvement of Sci-Tech and R & D efficiency. This may be because the three provinces have a poor level of economic development and do not pay enough attention to the R & D and innovation of pharmaceutical manufacturing industry. Technological retrogression has limited the improvement of R&D efficiency, which may be owing to the poor level of economic development and insufficient attention to the R&D and innovation of pharmaceutical manufacturing industry. Liaoning (0.9123), Tianjin (0.9388), and Zhejiang (0.9812) in the eastern region have poor performance in RDS. Further analysis of the EC and TC shows that the efficiency of technological progress in the three provinces is < 1, which limits the improvement of research and development. While some provinces have high efficiency, other provinces have negative efficiency growth. The negative growth in some provinces reveals that the pharmaceutical manufacturing industry in China has the characteristics of “extensive,” so management should be strengthened, innovation ability should be improved and innovation resource allocation should be optimized.

### Research on the EETS

#### Research on the EETS from temporal perspectives

**Figure 6:**
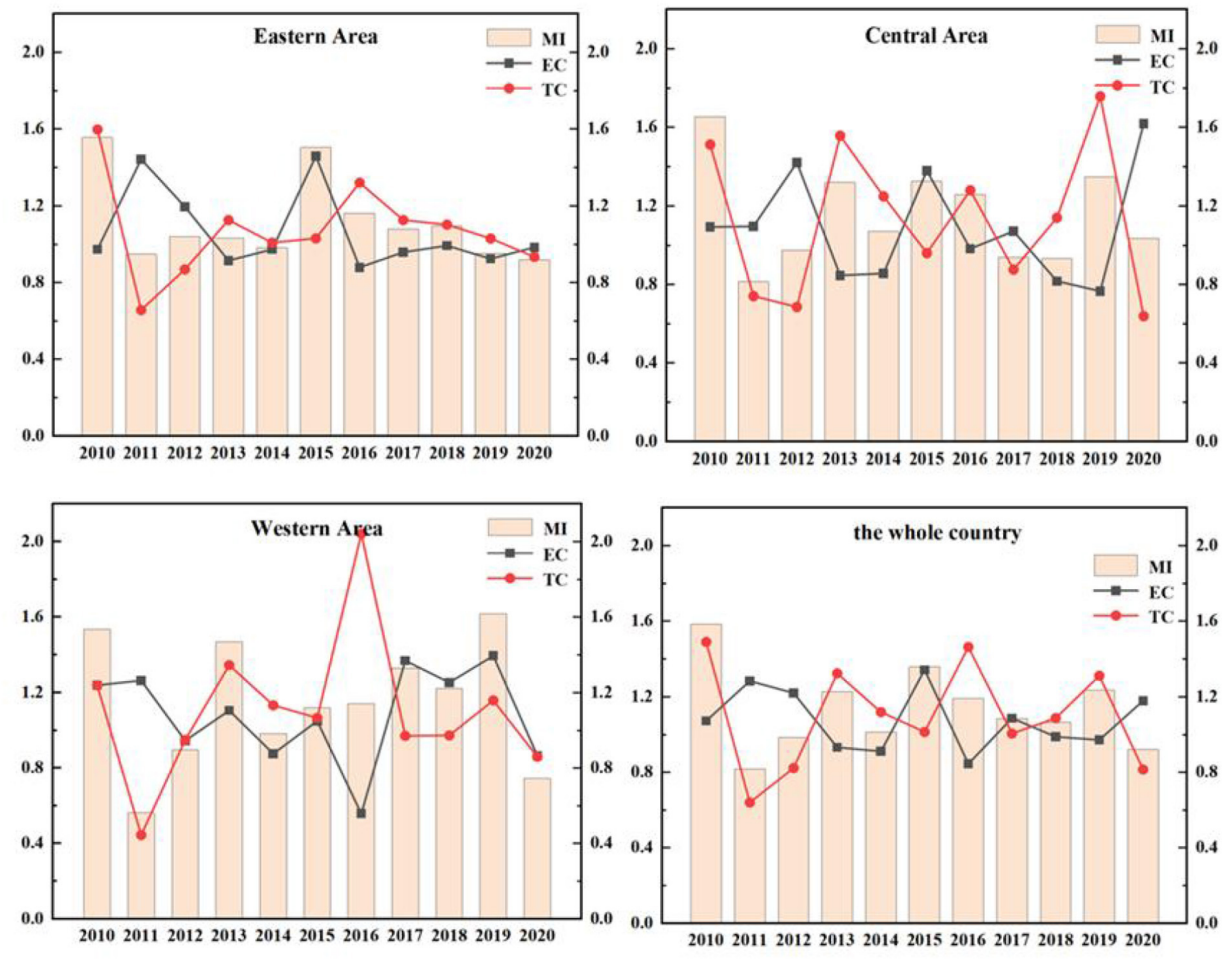
EETS and its decomposition index in different regions from 2010 to 2020.

The EETS reflects the ability of transforming R&D patent input into economic output. Due to the differences in local policies, economic distribution levels and traffic factors, the economic transformation ability of different regions is also different. As in Figure 6, the EETS of the eastern region, the central region and the western region fluctuates, which reflects that the economic transformation capacity of Chinese pharmaceutical manufacturing industry is very unstable.

The EETS of the eastern region from 2010 to 2020 was 1.5557, 0.9483, 1.0394, 1.0309, 0.9817, 1.5038, 1.1618, 1.0791, 1.0949, 0.9526, and 0.9195, respectively. The data showed fluctuations. In 2011, 2014, 2019, and 2020, the TFP was < 1, the EETS declined. The total factor productivity in other years was >1, and the economic transformation capacity was enhanced. During this period, the EETS was the highest in 2010, reaching 1.5557, and the technological progress index was 1.5981, >1, indicating that technological progress improved the EETS.

In the central region from 2010 to 2020, the years with EETS < 1 were as follows: In 2011 (0.8132), 2012 (0.9748), 2017 (0.9389), and 2018 (0.9321), the economic transformation capacity declined, and the TC in 2011, 2012, and 2017 was < 1. It showed that the decline of economic transformation capacity in these 3 years was mainly caused by technological regression. While the TC of 2018 was >1, its efficiency progress index was 0.8174, less than 1, leading to the reduction of EETS.

The TFP of the western region in 2010, 2012, 2014, and 2020 were 0.5633, 0.8779, 0.9829, and 0.7448, respectively, which were all < 1. These indicated a reduction in the EETS in these 4 years, which can be increased by 43.67, 12.21, 17.10, and 25.52%, respectively compared with the optimal distance level. Further studies on the decomposition index of the EETS in these 4 years showed that the TC in 2011, 2012, and 2020 were < 1. The main reason for the retrogression of economic transformation capacity was the retrogression of technological changes. The total factor productivity in other years was >1, which means that the economic transformation capacity has improved.

During this period, the efficiency of the national EETS showed fluctuations, and there was a brief decline in 2011, 2012, and 2020. In 2015, the national EETS reached the peak (1.3580). This phenomenon is mainly caused by two reasons. On the one hand, the intensification of aging in China led to the increase of medical demand. At the same time, with the improvement of the medical security system, the burden of medication on residents was reduced. Therefore, the income of the medical manufacturing industry increased. On the other hand, the government has been regulating drug prices in recent years. The pharmaceutical manufacturing industry has been under great pressure in the market, and the ability of economic transformation would also be declined temporarily.

**Figure 7:**
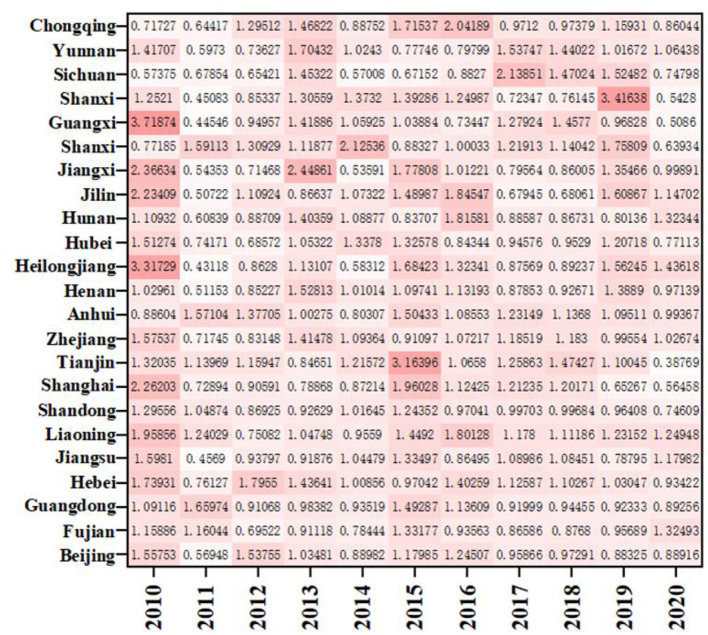
EETS in different provinces from 2010 to 2020.

As shown in Figure 7, the average distribution of EETS in eastern region, western region and central region is 1.1152, 1.1514, and 1.1475, all >1, indicating that the average level of economic transformation has been improved. The EETS firstly climbed up and then declined in the eastern region. Among them, the EETS of Sichuan from 2010 to 2020 is 0.5738, 0.6785, 0.6542, 1.4532, 0.5701, 0.6715, 0.8827, 2.1385, 1.4702, 1.5248, and 0.7480, respectively, with a large fluctuation range, indicating that the economic transformation capacity during this period is not very stable. Except for 2010 and 2015 to 2018, Shanghai's economic transformation index is >1. The economic transformation indexes were < 1 in all the other years. There was also a phenomenon of fluctuation. It means that the economic transformation capacity of the other years except the period is regressive, which may be because it takes 10 years for a drug clinical trial from the application to the successful listed. When the drugs listed, the sales will be affected by the factors such as drug approval, price and national policy to a certain extent. At the same time, some drugs in a certain stage of clinical trials are found that there are risks or unsolvable problems lead to clinical failures, leading to a low EETS in these regions in a certain period.

#### Research on the EETS from spatial perspective

**Figure 8:**
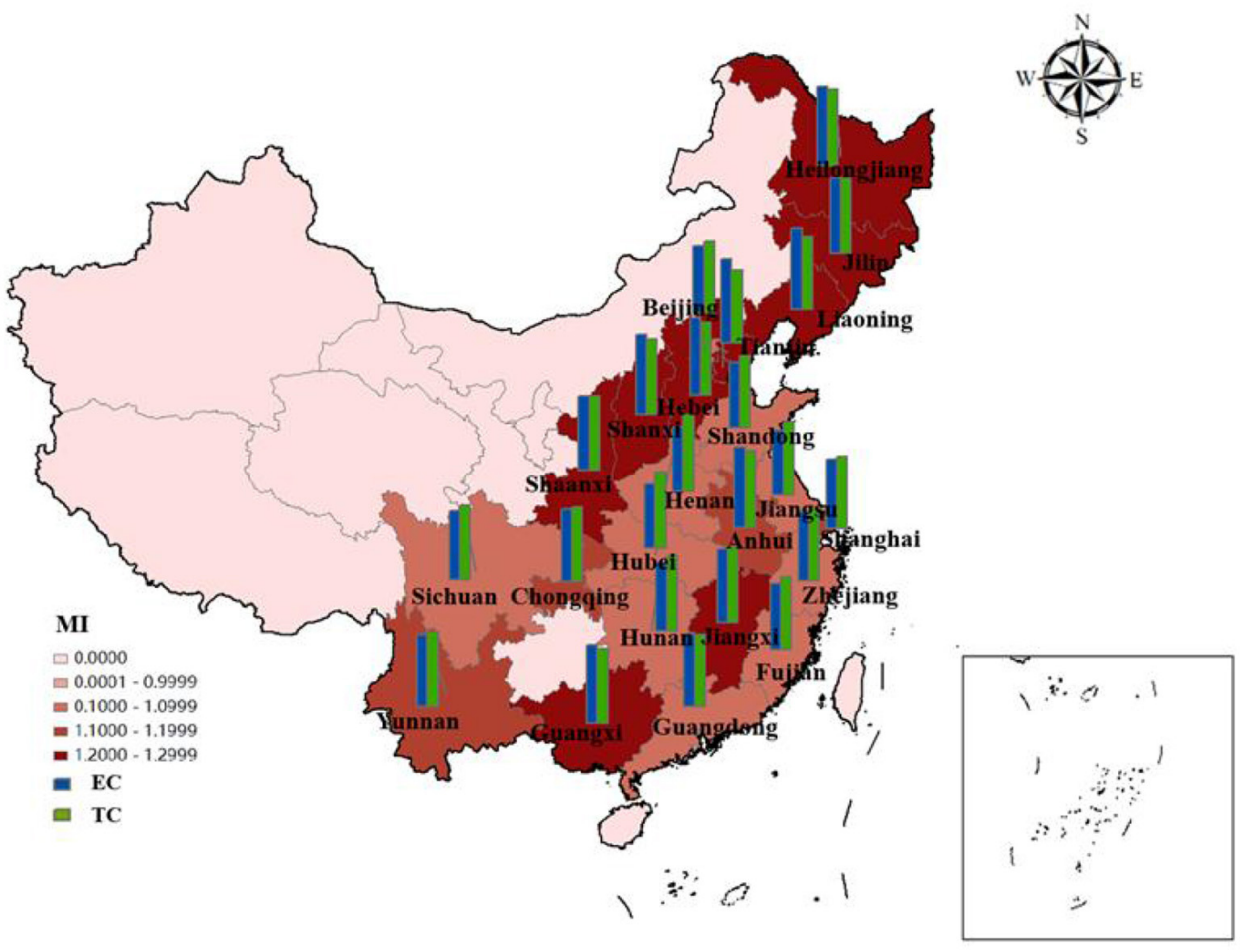
Average efficiency and its decomposition index of EETS in 23 Chinese provinces from 2010 to 2020.

As shown in Figure 8, the EETS of ten provinces in the eastern region is all >1, achieving progress in EETS. Among them, the EETS of Fujian is 1.0002, its technological progress index is 1.0731, but the efficiency progress index is 0.9801, which is < 1. Therefore, if Fujian wants to further improve the EETS, strengthening efficiency and progress will bring better development space.

In the central region, EETS increased by 15.14%, efficiency progress contributed 8.64%, and technological progress contributed 7.31%. Heilongjiang's EETS is 1.2818, which is developing well, even better than Shanghai. There may be two reasons: Firstly, most of the pharmaceutical innovations in Heilongjiang are relatively conservative innovations, and most of the patents obtained are relatively easy to be accepted. On the other hand, Shanghai, with its high level of internationalization and frequent high-level academic exchanges, can get in touch with the problems being solved in the world. The research and development cycle is different. It takes 8–10 years for some R&D patents to be successfully listed on the market. Therefore, the efficiency will be different. Although the efficiency is slightly less, it still has research value and significance. Secondly, most pharmaceutical companies in Shanghai will expand their overseas business and establish subsidiaries overseas, which will lead to the results of economic output not in the place of registration of the company and reduce the economic transformation ability to a certain extent.

In the western region, Guangxi has the highest EETS (1.2345), and its EC and TC are 1.1514 and 1.1079 respectively. The improvement of technical efficiency and technological progress jointly promote the improvement of economic transformation capacity. The economic transformation efficiency of Sichuan is 1.0332, which is lower than that of other western regions.

### Comparison between the ERDS and EETS

**Figure 9:**
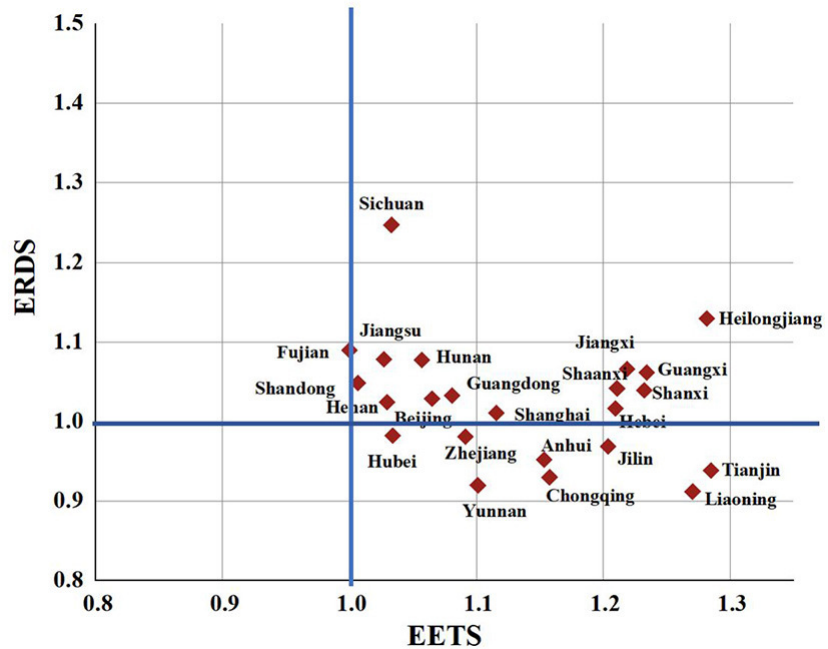
Clustering analysis chart of ERDS and EETS in provincial pharmaceutical manufacturing industry.

To better understand the differences in the innovation efficiency of the pharmaceutical manufacturing industry in 23 provinces, this paper will make a cluster analysis of the ERDS and EETS in each region. Taking 1.0 as the boundary, it can be divided into four types: A, B, C, and D, as in Figure 9.

Class A are regions with high ERDS and high EETS, both of which exceed 1.0, including Beijing, Fujian, Guangdong, Hebei, Jiangsu, Shandong, Shanghai, Henan, Heilongjiang, Hunan, Jiangxi, Shanxi, Guangxi, Shaanxi and Sichuan. The innovation efficiency in these regions hardly needs to be improved. However, Heilongjiang Province and Shaanxi Province may not be “high” innovation in the real sense, because they have less input, and relatively less redundancy and waste, which improves the output efficiency to a certain extent.

Class B are regions with high ERDS and low EETS. The 23 provinces studied in this paper are not included in such type.

Class C are regions with high ERDS and low EETS. The 23 provinces studied in this paper are not included in such type.

Class D are with low ERDS and high EETS, including Liaoning, Tianjin, Zhejiang, Anhui, Hubei, Jilin, Yunnan and Chongqing. In these regions, the ERDS is < 1, and the EETS is more than 1. It shows that the R&D and economic transformation activities in these regions are not coordinated. The low R & D efficiency limits the improvement of the efficiency of the regional pharmaceutical manufacturing industry. Therefore, the redundancy and waste should be reduced and the rationality of resource allocation should be improved in the pharmaceutical manufacturing industry in these regions while improving the R & D scale and strengthening technological innovation.

## Conclusions and policy recommendations

Based on the super network SBM model and the Global-Malmquist index, a study of pharmaceutical manufacturing's innovation efficiency of Chinese 23 provinces is presented in this paper from 2010 to 2020. The results show that:

(1) ERDS: In temporal perspectives, the development of ERDS is volatile. This could be due to different research and development cycles, as well as volatility caused by the difficulty and high risk of developing key technologies. In spatial perspective, The R&D efficiency of some provinces of the three regions is < 1, which is lower than the national average level. Technological regression limits the improvement of R&D efficiency. The negative growth in some provinces reveals that the pharmaceutical manufacturing industry in China has the characteristics of “extensive”. Therefore, the management should be strengthened, innovation ability should be improved, and innovation resource allocation should be optimized.
(2) EETS: In temporal perspectives. The development of the EETS of the pharmaceutical manufacturing industry is relatively volatile in China. In different regions, the EETS is different because different pharmaceutical patents take different time period to go through from clinical to market, and the market acceptance of new drugs is also different. In spatial perspective, the development of the three regions is generally good, and technological progress drives the improvement of EETS.
(3) By comparing the ERDS and EETS, it is found that Liaoning, Tianjin, Zhejiang, Anhui, Hubei, Jilin, Yunnan and Chongqing have weak R&D capabilities, but strong economic transformation capabilities. It found that the R&D of science and technology in these regions is not coordinated with the economic transformation, and the low R&D efficiency limits the improvement of the efficiency of regional pharmaceutical manufacturing industry.

The Chinese pharmaceutical manufacturing industry's innovation capability needs to be continually strengthened, based on empirical analysis, which is of great vital to the health of the people and the sustainable development in China. Taking into account China's actual pharmaceutical manufacturing situation, we recommend the following policies:

(1) In the research and development stage, if we want to improve the ERDS in the eastern, central and western regions, we should focus on improving technical efficiency, knowledge innovation efficiency and R&D intensity. Focusing on regional resources, different regions have their own unique resources. For example, the central region is rich in traditional Chinese medicine resources, which can be vigorously developed the traditional Chinese medicine industry, and the western region has traditional medicine with ethnic characteristics, which can be inherited and innovated at the same time. In the meantime, due to the long R&D cycle and high risk of new drugs, the government can provide special funding for some new drug research and development projects with high technology content and good development prospects.
(2) In the economic transformation stage, for the regions with low economic transformation efficiency in central and western regions, the efficiency and utilization rate should be further improved. It is important to optimize resource allocation, market structure should be improved, and the guiding role of markets should be emphasized in resource allocation and economic transformation. For the economically developed coastal areas in the east, the improvement of efficiency is mainly due to technological progress. In order to achieve better development, the efficiency utilization efficiency should be improved on the basis of maintaining the current technical efficiency. And the same time we should actively expand overseas markets and accelerate the sustainable and balanced development of China's pharmaceutical manufacturing.

## Data availability statement

Publicly available datasets were analyzed in this study. This data can be found here: The data is available from “China High-tech Industry Statistical Yearbook,” which is public and free. https://data.cnki.net/yearbook/Single/N2022010268.

## Author contributions

Conceptualization and software: SZ, FW, and SL. Methodology: YZho, YZhe, and SL. Data curation and writing-original draft preparation, writing-reviewing, and supervision: SL. All authors contributed to the article and approved the submitted version.

## Funding

This research was funded by the Heilongjiang Philosophy and Social Sciences Project (21JYE396 and 21JLE321) and Natural Science Foundation of Heilongjiang Province (LH2019H074).

## Conflict of interest

The authors declare that the research was conducted in the absence of any commercial or financial relationships that could be construed as a potential conflict of interest.

## Publisher's note

All claims expressed in this article are solely those of the authors and do not necessarily represent those of their affiliated organizations, or those of the publisher, the editors and the reviewers. Any product that may be evaluated in this article, or claim that may be made by its manufacturer, is not guaranteed or endorsed by the publisher.
